# Supplementary material for: MetaRibo-Seq measures translation in microbiomes
Source: Nat Commun. 2020 Jun 29;11:3268. doi: 10.1038/s41467-020-17081-z (PMC7324362; doi:10.1038/s41467-020-17081-z)
Supplement: Supplementary file 10 — Supplementary Data 7 [file 41467_2020_17081_MOESM10_ESM.zip › File2/Confidence_High_Taxonomy/109427_out.krona.html]

Javascript must be enabled to view this page.

members
magnitude
magnitudeUnassigned
count
unassigned
taxon
rank

109427\_out

4

superkingdom
4
2

phylum
1239
4

class
4
186801

4
186802
order

family
31979
4

genus
1485
4

4
1262781

SRS013476\_contig\_number\_contig-100\_1400.230579SRS024132\_contig\_number\_contig-100\_2009.235462SRS050752\_contig\_number\_25038SRS1041039\_contig\_number\_contig-100\_470.156501
species
